# Primary small cell carcinoma after renal transplant: A case report

**DOI:** 10.1097/MD.0000000000012592

**Published:** 2018-12-10

**Authors:** Xueli Wang, Fenghua Wang, Yumei Liang, Wen Chen

**Affiliations:** Department of Pathology, The 8th Medical Center of Chinese PLA General Hospital, Beijing, China.

**Keywords:** immunosuppressive therapy, renal transplant, small cell carcinoma

## Abstract

**Introduction:**

Primary small cell carcinoma (SCC) after renal transplantation is very rare. Here, we reported 1 case of primary SCC after renal transplantation and analyzed its clinical and pathological characteristics.

**Case presentation:**

A 55-year-old female underwent renal transplantation in our hospital 2 years ago and had been using tacrolimus for immunosuppressive therapy. Because of abdominal distention, the patient was admitted to our hospital. Computed tomography (CT) showed a malignant tumor of left kidney. Patient underwent surgical treatment and radical nephrectomy and lymph node dissection were selected. Postoperative pathological diagnosis was primary renal parenchyma and ureteral SCC. The patient has been treated with combination chemotherapy of lowpol (100 mg per day) and etoposide (10 mg per day). His vital signs are stable now, and he is receiving further treatment in our hospital.

**Conclusion:**

Because of immunosuppressive drugs use, the incidence of malignancies has increased significantly after renal transplantation. This case highlights the difficulty of diagnosis of primary SCC and the necessity of checking for neuroendocrine tumor after organ transplantation.

## 1 Introduction

Small cell carcinoma (SCC) occurs mainly in the lungs. Extrapulmonary SCC accounts for 2.5% to 5.0% of all SCC and their main sites are bladder, prostate, gastrointestinal tract and cervix.^[1]^ Primary renal SCC is rare, primary SCC after renal transplantation is more rare.^[2,3]^ Here, we reported 1 case of primary SCC after renal transplantation and analyzed its clinical and pathological characteristics.

## 2 Case report

**Figure 1:**
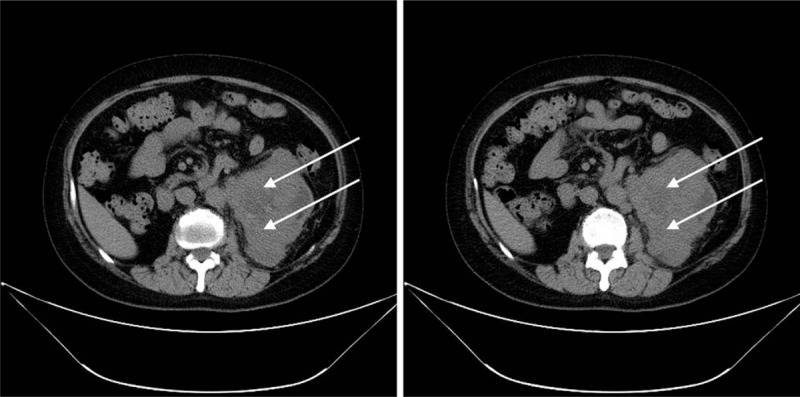
Computed tomography (CT) showed a malignant tumor of left kidney. CT = computed tomography.

This study was approved by the Ethics Committee of 309th hospital of PLA and was performed according to the principles of Declaration of Helsinki. The patient has provided informed consent for publication of this case, and the ethics committee approved the consent procedure. A 55-year-old female underwent renal transplantation in our hospital 2 years ago and had been using tacrolimus for immunosuppressive therapy. Because of abdominal distention and abdominal mass, the patient was admitted to our hospital recently, and computed tomography (CT) showed a malignant tumor of left kidney. The CT showed that left kidney had irregular mass with a diameter of about 9.3 cm, showing low density necrotic foci and a few punctate calcifications (Fig. 1). Radiologists considered the possibility of renal pelvis carcinoma or urothelial carcinoma.

Patient underwent surgical treatment, and radical nephrectomy and lymph node dissection are selected. After surgery, patient has been treated with combination chemotherapy of lowpol (100 mg per day) and etoposide (10 mg per day). Hemodialysis is used to replace kidney function. This patient has been followed up for 8 months after surgery. His vital signs are stable now, and he is receiving further treatment in our hospital.

Left kidney and part of ureter tissues were examined, and the size of kidney was 14 × 9 × 7 cm. A gray white mass can be detected near the renal pelvis, and its size was 10 × 7 × 6 cm. The cutting surface is gray and medium. Another gray white mass appeared in the ureter cavity, 6 × 3.5 × 2.5 cm in size. Some lymph nodes were found in the hilum, and the diameter was 0.3 to 1.4 cm.

**Figure 2:**
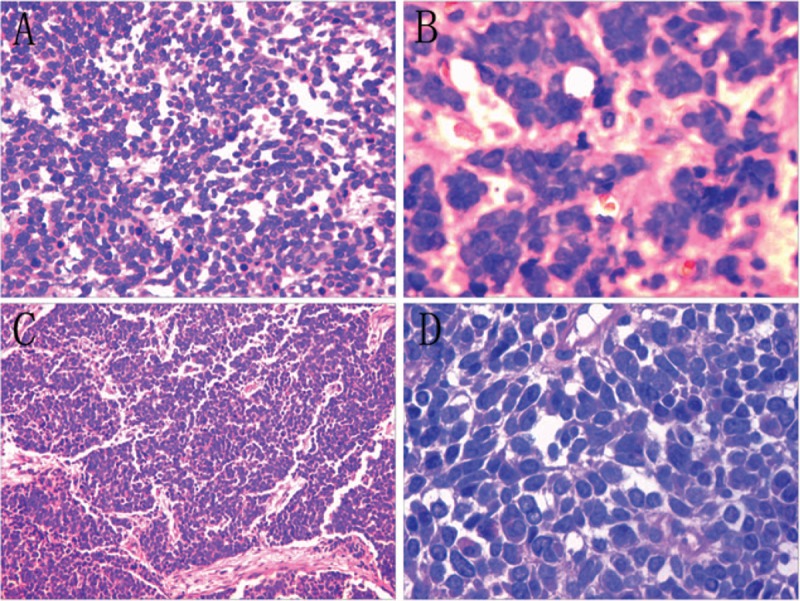
HE staining. (**A**) Renal SCC (100×); (**B**) Renal SCC (400×); (**C**) Ureter SCC (100×); (**D**) Ureter SCC (400×). SCC = small cell carcinoma.

Tumor consisted of short spindle and small blue round cells. The tumor cells were nested and trabecular arranged, with large foci of necrosis and hemorrhage. Nucleus was deeply dyed, and nuclear division was more common. Tumor tissues grew diffusely and were extensively infiltrated the surrounding renal medulla, cortex, renal pelvis and adipose tissue around the renal hilum. Tumor suppository was formed in pulse tube. Metastatic small cell carcinoma nests can be observed in hilar lymph nodes (Fig. 2).

**Figure 3:**
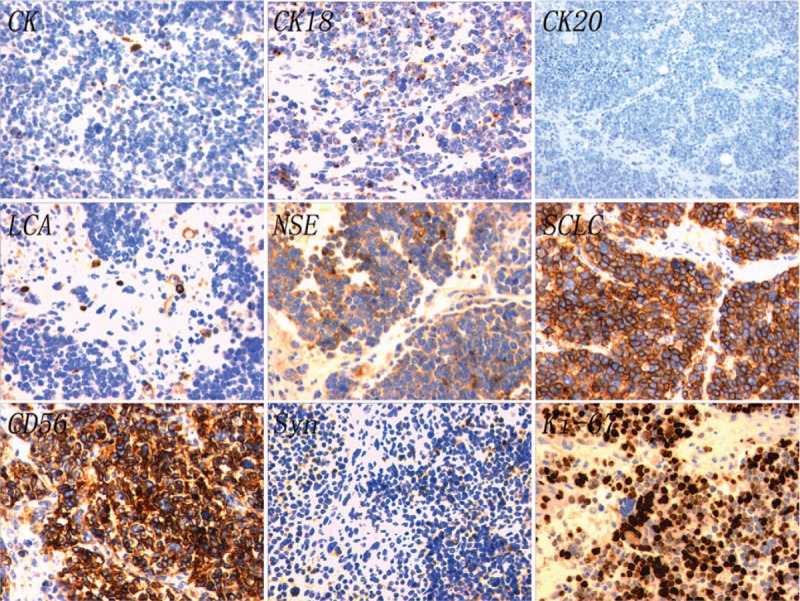
Immunohistochemical staining. CK (+), CK18 (+), CK20 (−), LCA (a few +), NSE (++), SCLC (++), CD56 (++), Syn (a few +), Ki-67 (+ > 75%).

Immunohistochemical results were as follows. Positive expression: CK (+), CK18 (+), CK5/6 (a few +), CD56 (++), NSE (++), SCLC (++), Syn (a few +), LCA (a few +), CD34 (blood vessel +), P40 (a few +), p63 (a few +); Negative expression: CD138 (−), CD38 (−), CK20 (−), CK7 (−), CgA (−), S-100 (−), Vimentin (−), CD10 (−), Pax-8 (−); Proliferation index: Ki-67 (+ > 75%) (Fig. 3).

To sum up, the patient's pathological diagnosis was primary renal and ureteral SCC with obvious hemorrhage and necrosis. Tumor thrombus was found in the vessel. Tumor cell infiltrated ureter into the serosa, invaded focal pelvis, renal sinus fat, renal capsule and perirenal fat capsule. Tumor metastasis (3/7) can be observed in renal hilar lymph nodes.

## 3 Discussion

Because of immunosuppressive drugs use, the incidence of malignancies has increased significantly after renal transplantation.^[4]^ It is generally believed that there are 3 main sources of tumor after renal transplantation: primary tumor after renal transplantation; tumor recurrence before renal transplantation; tumor derived from donor kidney. Urinary tract tumor especially urothelial carcinoma, is the main type of tumor after renal transplantation.^[5]^

The proportion of neuroendocrine carcinoma in renal epithelial malignancies is less than 1%. Renal small cell carcinoma (SCC) is a special neuroendocrine carcinoma, which is relatively rare. Since the 1st report of renal SCC in 1984, dozens of cases have been reported in papers. However, primary SCC in transplanted kidney is rarely reported.^[6,7]^ The clinical symptoms of renal SCC are mainly abdominal mass, abdominal pain and gross hematuria. The peak age is mostly middle-ages and elderly, and women are more common than men.

Extrapulmonary SCC can occur in many organs, including ovary, cervix and pancreas. In urogenital organs, the most common primary site is bladder, followed by prostate, kidney, and ureter.^[8]^ The common clinical symptoms were painless gross hematuria, followed by waist and abdominal pain.^[1]^

Renal SCC can occur in renal cortex, medulla and renal pelvis. Because their symptoms are not obvious, the tumor is very large and often metastases to lymph nodes when patients come to the hospital. The tumor is composed of poorly differentiated small blue round cells or short spindle cells. Tumor cells are arranged in small nests or trabeculae, often accompanied by necrosis and hemorrhage. The nuclei of tumor cells are rich in chromatin, nucleolus is not obvious, cytoplasm is sparse, mitotic figures are abundant, and mitotic index is high. There are many tumor thrombus formation in the vessels, and some patients with renal SCC may also have urothelial carcinoma. Immunohistochemical staining shows that tumor cells have strong positive expression on neuroendocrine markers, and most of them express epithelial markers. In this case, renal SCC had all above characteristics. Immunohistochemical staining showed that tumor cells expressed multiple epithelial and neuroendocrine markers simultaneously.

The origin of renal SCC is still unclear. Some studies suggested that the SCC of urogenital system may originate from the pluripotent stem cells of urogenital tract. Renal SCC can also be accompanied by urothelial carcinoma, squamous cell carcinoma and adenocarcinoma, which may support the hypothesis that the tumor originates from ureteral pluripotent stem cells.^[9,10]^ Some researchers believed that tumors originate from the normal neuroendocrine cells in genitourinary tract. This view is supported by the expression of neuroendocrine markers in renal SCC.^[11,12]^ Further studies are needed to elaborate its pathogenesis and origin.

Renal SCC is easily misdiagnosed as other small cell tumors, such as undifferentiated carcinoma, Ewing tumor, embryonal rhabdomyosarcoma, lymphoma and primitive neuroectodermal tumor. Immunohistochemistry and electron microscopy are helpful for differential diagnosis. Renal SCC usually has more than 2 neuroendocrine markers (NSE, CD56, Syn, CgA, CD99, etc.) positive expression.^[13–15]^ We should pay attention to the possibility of external metastasis if we diagnose primary renal small cell carcinoma. Poorly differentiated urothelial carcinoma mainly expresses epithelial markers and does not express neuroendocrine markers.^[16]^ Primary lymphoma of the ureter is rare, mainly expressing B lymphocyte markers, such as CD20, CD79a and so on. Metastatic renal SCC is also rare, and preoperative imaging examination helps to exclude the diagnosis.

Surgical resection is the 1st choice of renal SCC.^[17,18]^ Their prognosis is very poor, with an average survival time of 10.5 months. In this case, the tumor volume is very large, and radical nephrectomy and lymph node dissection are selected.

## Author contributions

**Conceptualization:** Fenghua Wang.

**Data curation:** Xueli Wang, Wen Chen.

**Investigation:** Xueli Wang, Fenghua Wang, Yumei Liang.

**Methodology:** Yumei Liang.

**Writing – original draft:** Wen Chen.

**Writing – review & editing:** Wen Chen.
